# Gut-Brain Axis and its Neuro-Psychiatric Effects: A Narrative Review

**DOI:** 10.7759/cureus.11131

**Published:** 2020-10-24

**Authors:** Likhita Shaik, Rahul Kashyap, Sahith Reddy Thotamgari, Romil Singh, Sahil Khanna

**Affiliations:** 1 Internal Medicine, Ashwini Rural Medical College, Hospital & Research Centre, Solapur, IND; 2 Medical Oncology, Mayo Clinic, Rochester, USA; 3 Anesthesiology, Mayo Clinic, Rochester, USA; 4 Cardiology, Mayo Clinic, Rochester, USA; 5 Internal Medicine, Louisiana State University Health Sciences Center, Shreveport, USA; 6 Internal Medicine, Metropolitan Hospital, Jaipur, IND; 7 Gastroenterology, Mayo Clinic, Rochester, USA

**Keywords:** gut-brain axis, microbiome, neuropsychiatric, parkinson, alzheimer’s, tmao

## Abstract

The gut microbiota regulates the function and health of the human gut. Preliminary evidence suggests its impact on multiple human systems including the nervous and immune systems. A major area of research has been the directional relationship between intestinal microbiota and the central nervous system (CNS), called the microbiota-gut-brain axis. It is hypothesized that the intestinal microbiota affects brain activity and behavior via endocrine, neural, and immune pathways. An alteration in the composition of the gut microbiome has been linked to a variety of neurodevelopmental and neurodegenerative disorders. The connection between gut microbiome and several CNS disorders indicates that the focus of research in the future should be on the bacterial and biochemical targets. Through this review, we outline the established knowledge regarding the gut microbiome and gut-brain axis. In addition to gut microbiome in neurological and psychiatry diseases, we have briefly discussed microbial metabolites affecting the blood-brain barrier (BBB), immune dysregulation, modification of autonomic sensorimotor connections, and hypothalamus-pituitary-adrenal axis.

## Introduction and background

The human gut harbors a diversity of microbiome which interact with the human host [1]. It is inhabited by approximately 10^13^-10^14^ bacteria predominated by bacterial phyla like the Firmicutes, Bacteroidetes, Actinobacteria, Proteobacteria, Fusobacteria, and Verrucomicrobia [2,3]. The gut microbiota is also based on the modality of birth. After birth, many factors play a role in modifying it. These factors include diet, infections, antibiotic use, host genetic factors, age, amongst others [4]. The increasing evidence to endorse the influence of the enteric microbiome on the brain function through various neurotransmitters indicating its crucial role in modulating host cellular mechanisms, which are essential in many neuropsychiatric conditions. [5]. Many studies were successfully associated with the gut microbiota with various neurological and psychiatric illnesses [6-10].

Although ample research was conducted for trying to uncover the nature of the relationship that links the gut microbiota and the Brain, several questions remain unanswered. More insight into the mechanism of communication may give rise to the prospects of intestinal flora-based remedies, which can assist in the treatment of central nervous system (CNS) disorders. This area provides a field for translation of basic research to clinical practice. Firstly, use of the human microbiome to estimate the risk of neurological disease and secondly, to develop further research to use the microbiome as a therapeutic target, because the microbiome content in a human body is modifiable by factors like diet, environment, and pharmaceuticals.

## Review

The mechanism and various factors that link the interaction between the gut and the brain has been studied widely since the last decade, of which the most prominent mechanisms described below can be majorly attributed to the impact [11]. The gut-brain axis comprises the brain, endocrine system, immune system, autonomic outflow, and the gut microbiome [12].

Microbial metabolites affect the blood-brain barrier (BBB)

In the circulation of healthy individuals, hundreds of perceptible microbial metabolites have been identified to be associated with intestinal dysbiosis [13,14]. Gut derived microbial metabolites like lipopolysaccharides (LPS) and fatty acids such as butyrate, propionate, and acetate affect the permeability of the BBB [15].

Immune dysregulation

There is a strong relationship between the gut microbiome and immune system; there have been studies relating to multiple sclerosis and optic neuritis (which are autoimmune diseases) to gut microbiome [11]. Cytokines released by cell-mediated immunity, like IL-6, TNF-alpha, IL-1b, play a role in disorders like depression where these cytokines can act as risk factors or aggravate the state of depression [16].

Modification of autonomic sensorimotor connections: vagus nerve and neurotransmitters

The vagus nerve lies at the interface between the CNS and the gut [17]. It detects changes in the gut microbiota, which translates and integrates into the CNS, leading to either an enhancement or deterioration in the functioning of the CNS [18,19]. Studies show that vagal nerve stimulation strengthens the expression of neurotrophic elements which advance the development of neurons which is implicated to be deranged in mood disorders [20]. Gut bacteria like E Coli, Proteus [21], Bacillus [22], Klebsiella [23], Bifidobacterium [24], Lactobacillus [25,26] have been implicated in affecting the CNS by impacting the production or response to neurotransmitters like catecholamines, serotonin, GABA.

Hypothalamus-pituitary-adrenal (HPA) axis

HPA axis is a crucial element of the limbic system that controls emotions and memory. [27] Studies indicate that the existence of microbiome is necessary for the transformation and growth of the HPA axis in early life, by way of expression of neurotrophic factors [11].

Various components of the bacterial cell wall like the peptidoglycan, lipopolysaccharides, and toll-like receptors are known to affect the permeability of the intestinal barrier in response to cortisol induced stress, which in turn activates the HPA axis, suggesting its role in the regulation of emotions and memory [28,29].

Gut microbiome in neurological diseases

The microbiome significantly influences articulation between the gut and the brain via the unified neuroendocrine and immunological processes. Gut microbiome dysbiosis has long been known to be associated with various derangements in brain function and cognition. Alzheimer's disease (AD) is a significant condition that has been studied in this aspect. Intestinal flora can regulate the host brain function and activity, including cognitive behavior, through the microbiome-gut-brain axis [30]. A large number of amyloids and other toxins produced by gut microbiota act as a source of systemic inflammation and undermine the physiological barriers, thus, playing a significant role in Alzheimer's disease [31]. Diet, antibiotics, probiotic interventions, gut microbiome disturbances leading to increased intestinal permeability, microbial metabolites are all known to affect the risk of AD. The role of gut microbial metabolites in the etiology of AD has been investigated in several studies. Small molecules such as trimethylamine N-oxide (TMAO), which are produced by metaorganismal choline metabolism, have been highlighted as risk factors for AD and significantly linked with the cognitive decline and age of onset of AD [32]. A study by Vogt et al. found the CSF TMAO to be higher in patients with mild cognitive impairment and AD and to be associated with biomarkers of AD pathology and neuronal degeneration [33].

It is also known that Parkinson's disease (PD) is accompanied by intestinal microbiota dysbiosis. The pathology of PD includes the enteric and central nervous systems, which are interconnected to each other via the vagus nerve. A study by Devos et al. suggested that gut inflammatory alterations in the Gut play a significant role in alpha-synuclein misfolding [34]. The gut microbiota alterations precede or occur during PD. Scheperjans et al. analyzed the fecal samples of PD patients and deduced that higher concentrations of Enterobacteriaceae were directly proportional to gait and postural instability [35]. Bedarf et al. found significant differences in colonic microbiota and microbiota metabolism involving B-glucuronate and tryptophan metabolism between PD subjects and healthy controls [36]. A study by Hill-Burns et al. showed reduced levels of lachnospiraceae, which produces short-chain fatty acids (SCFAs) in the gut. Hence consistent with the SCFA depletion in PD noted in previous studies [37]. 

Several studies have also examined the role of the gut microbiome in amyotrophic lateral sclerosis (ALS) and Huntington's disease (HD). In a recent survey with the ALS mouse model, a tight junction structure, greater intestinal permeability, and an altered microbiome profile with lower butyrate-generator bacteria were observed compared to controls. Butyrate, a bacterial metabolic by-product, has been proposed to normalize the gut microbiota, as well as to enhance the lifespan of ALS [38]. Thiamine, a by-product of bacteroidetes, is believed to play a role in the pathophysiology of HD, as thiamine supplements have been shown to benefit the debilitated energy metabolism at the cellular level. Hence, the therapeutic potential of microbiota modulation needs to be further explored [39].

Gut microbiota was proposed as an immune or inflammatory response booster as it plays a crucial role in fighting the infections. Van den Hoogen et al. suggested that autoimmune diseases, including multiple sclerosis, can be associated with digestive dysbiosis, systemic inflammation, and increased intestinal permeability [40]. Treatment aiming at the modification of gut microbiota may be an essential treatment of multiple sclerosis [41].

Role of gut microbiome in psychiatric diseases

Alterations to intestinal microflora and intestinal permeability have been studied about mental health, and general bacterial magnitudes are directly proportional to anxiety/cognition parameters [42]. The 'leaky gut,' which allows bacterial metabolites to cross the intestinal barrier, is implicated in Autism Spectrum Disorder. The presence of autistic symptoms was found to be correlated with less varied gut microbiome and excessive levels of Clostridium spp. and Desulfovibrio spp [43]. Aarts et al. examined the difference in gut microbiome between attention deficit hyperactivity disorder (ADHD) and their healthy controls. A significant rise in genus Bifidobacterium was found related to an improved biosynthesis ability of a dopamine precursor throughout the gut flora of ADHD patients, further correlating with the alteration in the reward anticipation responses in the brain, which is the neural hallmark of ADHD [44].

A 'leaky gut' provoked by starvation and increase in mucin degrading bacteria is characterized by antigens traversing through the intestinal wall. The antigens cause chronic low-grade inflammation, which is presumed to be present in anorexia nervosa. Morita et al. discovered a reduction in the percentage of total bacteria and obligate anaerobes in anorexia nervosa patients [45]. Armougom et al. found an increase in the amount of Methanobrevibacter smithii in anorexic patients in gut flora as compared to healthy controls and obese individuals [46]. Depression and anxiety symptoms found in AN patients are frequently more significant in those with gut dysbiosis [47]. Finally, among specific nutrients that could modulate the gut-brain axis in AN, omega-3 fatty acids and glutamine have emerged as exciting candidates with beneficial effects in maintaining intestinal barrier integrity [48].

Rett syndrome is also known to be associated with dysbiosis of both bacterial and fungal components of gut microbiota. This dysbiotic microbiota has been found to produce altered SCFA, which is suggested to reinforce the constipation status of these patients and contribute to the gastrointestinal pathophysiology in Rett syndrome [49].

**Figure 1:**
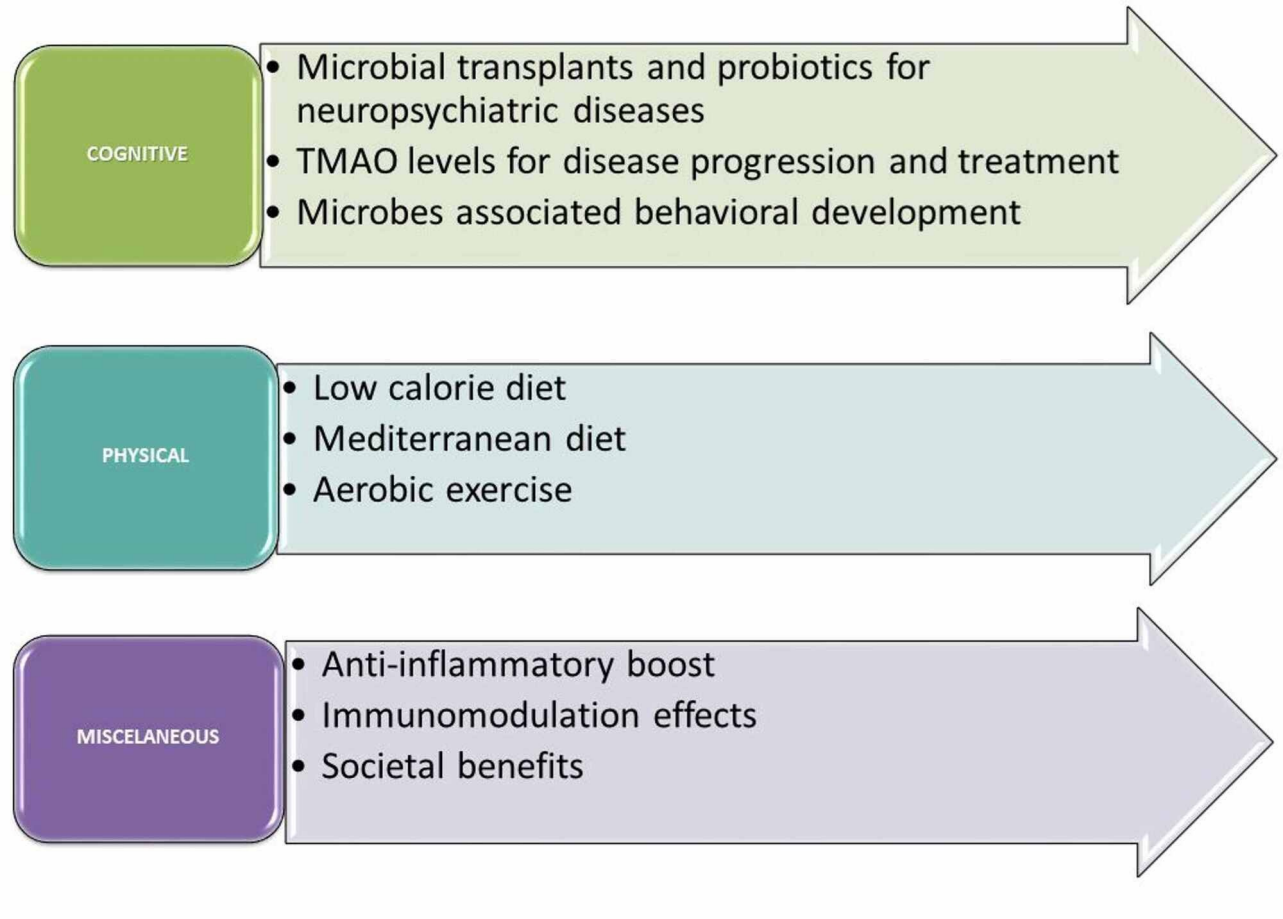
Neuro-psychiatric effects of gut-brian axis and recommendations TMAO, trimethylamine N-oxide

Recommendations (Figure 1):

Cognitive: 

1. Structural differences can be analyzed of gut microbiota between AD patients & healthy individuals to identify AD-specific microbes. Also, then we can transplant these microbes into gut microbiota depleted lab animals & study if this induced Alzheimer's disease. 

2. TMAO is a gut microbiome-based metabolite with high levels of Alzheimer's dementia patients. Long term studies are crucial to analyze whether high TMAOs levels in middle age predict continued development of Alzheimer's disease. Also, pharmacological drugs can be designed to inhibit excess TMAO production by the gut microbiome. Gut Microbiome based metabolite with high levels in Alzheimer's dementia patients. Long term studies are crucial to analyze whether high TMAOs levels in middle age predict continued development of Alzheimer's disease. Also, pharmacological drugs can be designed to inhibit excess TMAO production by gut microbiome. 

3. Microbial composition of Gut at one year of age predicts cognitive performance at two years of age, especially communicative behavior. Longitudinal studies incorporating multiple measurements would give a better idea of the temporal relationship between gut bacteria & brain development. 

4. Neuropsychological defects such as cognitive impairments and diminished neurogenesis in hippocampus have been demonstrated to develop in mice after the depletion of intestinal microbiota with antibiotics. Such impairments may be resolved by oral administration of probiotic bacteria. The effect of probiotics on depression & anxiety-like behavior needs to be studied in larger human samples. 

5. The relative proportion of Acidaminococcus, Enterobacteriaceae, Fusobacteriaceae, Porphyromonadaceae & Rikenellaceae were significantly higher in patients with active major depressive disorder compared to healthy controls while lower levels of Bacteroidaceae, Erysipelotrichaceae, Lachnospiraceae, Prevotellaceae, Ruminococcaceae & Veillonellaceae in functional major depressive disorder than healthy controls. The mechanism of how these microbiomes correlate with significant depression needs to be studied. 

Physical:

Inflammation reduction, improved epithelial membrane integrity, and increased microbial diversity were seen in recent studies on laboratory animals showing beneficial effects of aerobic exercise on the gut. Professional athletes are shown to have a wide variety of gut microbiome. More studies are certainly needed to confirm this beneficial effect on individuals. 

A high-fat diet leads to gut dysbiosis, while a low calorie & Mediterranean style diet may hold promise for balancing gut microbiome. 

Miscellaneous:

1. The gut microbiome has enhanced intestinal barrier function, antimicrobial, anti-inflammatory and immunomodulation effects 

Research should be oriented to pave a path towards welfare not only in the field of health but also in other sectors of society. For example: If studies found a correlation between criminal behavior and Gut microbiome impact, it could aid in preventing crimes by treating such actions.

## Conclusions

The microbiome is an integral part of its host's well-being. While many studies on this subject to date have shown that different bacterial populations are associated with several clinical conditions, it is relatively unknown whether such disparities play a role in the pathogenesis of various diseases. Through this literature review, we also conclude that it was difficult to establish a distinct pattern of the impact that gut microbiome has on brain with respect to geographical distribution. Such aspects of interaction between brain function and the microbiome complex relationship and should be continued to be addressed in future research. Also, we need to find out if the changes in the microbiome are the cause of its underlying pathophysiology, or are they a result thereof. The microbial features necessary for neurodevelopment and prevention of neurodegeneration need to be defined in further studies.
